# Effect of flower identity and diversity on reducing aphid populations via natural enemy communities

**DOI:** 10.1002/ece3.8432

**Published:** 2021-12-06

**Authors:** Sharon E. Zytynska, Moritz Eicher, Robin Fahle, Wolfgang W. Weisser

**Affiliations:** ^1^ Department of Evolution, Ecology, and Behaviour Institute of Infection, Veterinary and Ecological Sciences University of Liverpool Liverpool UK; ^2^ Terrestrial Ecology Research Group Department of Ecology and Ecosystem Management School of Life Sciences Weihenstephan Technical University of Munich Freising Germany

**Keywords:** aphid, cereal crop, flowering plant, indirect interactions, natural enemy, parasitoid, pest control, pest suppression, predator

## Abstract

Floral plantings are often used in agriculture to attract pollinator communities, but they also play an important role in recruiting and establishing natural communities for natural pest control. Inconsistent effects of floral plantings for pest control may be a result of an absence of mechanistic insights and a reliance on the idea that simply increasing flower diversity will benefit these services. A more tailored set of flower species may be needed to benefit the natural enemies through provision of nectar and alternative prey. We used an outside pot experiment to investigate the effect of three flower plants (*Fagopyrum esculentum*, *Vicia faba*, *and Trifolium pratense*) on reducing aphid pests on four different plant cultivars of barley (*Hordeum vulgare*), over two years. We grew the four cultivars of barley alone, next to a single flower or next to a mixture of flowers, and observed aphid and natural enemy colonization across the growing season. Aphid population sizes were reduced on all barley cultivars grown next to a flower with stronger pest suppression when all flowers were present. Each flower species recruited a different community of non‐barley aphids that, in turn, varied in their ability to establish the natural enemy populations and subsequently the ability to reduce barley aphid populations. Overall, increased pest suppression in the mixed treatments was a result of numerous weaker interactions between different flower, aphid, and natural enemy species, rather than a few dominant interactions. Natural enemy communities could be enhanced by incorporating flower species that vary in their ability to attract and host alternative prey (i.e., non‐pest aphids) as well as suitable nectar provisioning. We can use our knowledge of ecological interactions to tailor floral plantings to increase the effectiveness of pest control services.

## Graphical abstract

Floral plantings are often used in agriculture to attract pollinator communities, but they also play an important role in recruiting and establishing natural communities for natural pest control. We used an outside pot experiment to investigate the effect of three flower plants (*Fagopyrum esculentum*, *Vicia faba*, and *Trifolium pratense*) on reducing aphid populations on potted barley plants (*Hordeum vulgare*), over two years. Each flower species recruited a different community of nonbarley aphids that, in turn, varied in their ability to establish the natural enemy populations and subsequently the ability to reduce barley aphid populations.

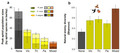

## 1 INTRODUCTION

Within the framework of Integrated Pest Management (IPM), understanding the ecology of the system is key to identifying the most suitable methods of pest control (Thomas, 1999). With estimated global yield losses of up to 40% from pests and pathogens (Savary et al., 2019), climate change‐driven pest range expansion (Bebber et al., 2013), evolution of pesticide resistance (Whalon et al., 2008), and recent bans on many environmentally damaging pesticides (Bakker et al., 2020), alternative solutions to pest control are needed. One commonly studied method is biological control by natural enemies, yet the effectiveness of this varies across systems and landscapes (Cohen & Crowder, 2017). In many agricultural systems, agri‐environment schemes (AES) involving flower strips, banker plants, and intercropping are used to increase key ecosystem services provided by biodiversity, including pest regulation and pollination (Albrecht et al., 2020; Bommarco et al., 2013; Lichtenberg et al., 2017). Floral plantings are often used to attract pollinator communities, but they also play an important role in recruiting and maintaining natural enemy communities that consume insect pests (Haenke et al., 2009), under the framework of conservation biological control (Heimpel & Mills, 2017). Many natural enemies directly benefit from plant nectar, as the adults feed on nectar while the larvae feed on insects; provision of a nectar source can increase a parasitoid wasps’ life span by up to 14.7‐fold and increase their host‐searching time from 3 days to 2 weeks (Russell, 2015). However, inconsistent outcomes of using floral plantings have hindered more widespread use (Albrecht et al., 2020; Hatt et al., 2020; Lowe et al., 2021).

In the absence of detailed mechanistic insights, it has been suggested that increasing diversity of plant species in flower strips, either species richness per se, or flower trait/functional diversity, can help to increase biocontrol (Balzan et al., 2014; Gurr et al., 2003). The argument is that increasing plant species richness is associated with increasing insect diversity including pest species natural enemies (Ebeling et al., 2018; Scherber et al., 2010) and that this increasing natural enemy diversity is associated with more effective biocontrol (Cardinale et al., 2003). In addition, a diversity of natural enemies will avoid selection for resistant pest populations; similar to resistance to pesticides, insects can also evolve resistance to specialized natural enemies either themselves or through interactions with microbial symbionts (McLean & Parker, 2020; Zytynska & Meyer, 2019). As a consequence, current flower mixtures for floral plantings often use a selection of local and native flower species that hope to provide various resources for increasing pollinators and natural enemy communities (Hatt et al., 2020). However, simply increasing flower species richness or plant functional diversity in fields does not necessarily translate into increased pest control (Albrecht et al., 2020; Hatt et al., 2017). Natural enemies are recruited and established on the noncrop flower plants (by nectar or alternative prey) but must spill over into the crop to consume the pest insects (Blitzer et al., 2012; Morandin & Kremen, 2013). If the increased plant diversity traps the natural enemies due to overabundant resources, the noncrop plants will compete with the crops for these services resulting in reduced crop pest control (Kremen et al., 2019). Similarly, nectar provision is important, but while nectar from many flowers enhances natural enemy host searching behavior, others have minimal effect (Bianchi & Wäckers, 2008; Russell, 2015). Thus, a more tailored combination of plants is required to recruit effective natural enemy communities (Tschumi et al., 2016).

Including a more mechanistic understanding of the ecology of pest and natural enemy species, such as their host ranges, may improve conservation biological control. Ideally, the noncrop plant would not host the pest insect (else it acts as a source or reservoir for new infestations) but rather host alternative prey insects that share a common natural enemy with the crop pest. Similar considerations can be taken for intercropping strategies. For example, the aphid *Aphis fabae* is a pest of beans and sugar beet yet does not feed on cereal crops, while cereal aphids (e.g., *Sitobion avenae*) do not feed on the beans or sugar beet (Blackman & Eastop, 2000). Intercropping beans (with their nitrogen‐fixing rhizobia) and cereals not only benefits nutrient cycling (Ofori & Stern, 1987) but also disease and pest reduction (Hansen et al., 2008; Zhang et al., 2019). This is because the host range of the natural enemies encompasses both groups of aphids.

Here, we investigated how species richness and the identity of different flowering plants can influence the recruitment and establishment of natural enemy communities (via flower or alternative prey resources). We aimed to elucidate mechanisms underlying aphid pest control under the framework of conservation biological control. Our outside model experimental system used individually potted barley plants (comparing effects across four barley cultivars that varied in aphid susceptibility in the laboratory) grown alongside the flower plants and allowed for natural colonization of all insect species. As flower plants, we used *Fagopyrum esculentum*, *Trifolium pretense*, and *Vicia faba* that have been used in flower strips, as banker plants, and have been previously used as cover or intercropping species with cereal crops. Further, both *Fagopyrum* and *Vicia* produce harvestable yields as an additional source of income for farmers (Yang et al., 2009), while *Trifolium*, as a nitrogen (N)‐fixing legume, benefits crop yield nitrogen fertilization of the soil (Thorsted et al., 2006). Lastly, there was no cross‐over of aphid species from these three flower plant species to the barley plants. We hypothesized that:

1. Increased flower richness reduces aphid populations via increased natural enemy diversity (aphid suppression)
2. Flower identity alters the strength of aphid suppression effects due to varying abilities to recruit and establish natural enemy populations
3. Aphid population growth varies across barley cultivars which in turn influences the effect of flower treatment and natural enemies on reducing aphid populations

## 2 METHODS

### 2.1 Study system

We used four cultivars of barley (*Hordeum vulgare* (Poaceae); Barke, Chevellier, Grace, Scarlett) and three other plant species that produce nectar‐bearing flowers (Buckwheat *Fagopyrum esculentum* (Polygonaceae); red clover *Trifolium pratense* (Fabaceae); and broad bean *Vicia faba* (Fabaceae)). The flowering plants were chosen based on criteria including (1) producing flowers within a suitable timeframe, (2) producing nectar that benefits parasitoid wasp (Russell, 2015), and (3) hosting different species of aphid to barley plants (i.e., no aphid community overlap). In addition, *F*. *esculentum* and *V*. *faba* have previously been intercropped with cereals, and all species have been used in agri‐environment flower strips to support pollinators and beneficial insect populations.

### 2.2 Experimental design

Five barley‐flower treatments were replicated across the four barley cultivars [20 treatments: individual potted barley cultivars alone, barley + *Vicia*, barley + *Trifolium*, barley + *Fagopyrum*, barley + *Vicia* + *Trifolium* + *Fagopyrum*] and each treatment combination replicated six times (120 barley plants grouped next to the flower plants). Using an additive randomized complete block design, each block contained one replicate of all treatments in a random order (4 × 5 design). Each plant (barley cultivar or flower species) was grown within its own individual 2‐L pot (i.e., no sharing of soil or water), with 1–4 pots grouped together depending on the treatment, the experimental area was bordered on one side by a small building and a meadow on the other. The barley plant was always placed toward the meadow side to avoid physical barriers to insects from the flowering plant. There was a minimum of 0.5 m between groupings of the pots within a single treatment and 1.5 m between blocks. The same experimental design was used in years 2017 and 2018, except the order within blocks was re‐randomized.

### 2.3 Experimental setup

Plants were germinated and grown in standard potting soil (Floragard B Pot Medium‐Coarse, pH 5.6, NPK 1‐0.6‐1.2) with no added fertilizer. In 2017, the plants were grown for 3 weeks inside a plant growth room (18°C 16:8 L:D), while in 2018, the plants were grown outside under a rain cover and mesh to avoid insect/rodent damage before the start of the experiment. The plants were placed outside on the 16th May 2017 and 29th May 2018 (delayed due to bad weather) and allowed to grow until harvest 60 days later (mid‐end of July). The plants were exposed to all weather conditions, and all insects could colonize. The plants were watered when needed (daily during warmer summer days) or excess water removed from pot saucers after rain. As the plants grew larger, sticks were used to support the *Fagopyrum* plants but no other plants needed the extra support.

### 2.4 Data collection

In 2017, data were collected twice weekly, whereas in 2018, this was done once weekly. The weather for the experiment was similar for the two years, but with higher initial temperatures in 2018 due to a delayed start from storm in the week before and periods of high rainfall on days 14 and 30 in 2018 (local weather data accessed from Bavarian State Research Centre for Agriculture). The plant variables collected were plant height (cm), flower number (individual flowers for *Vicia* and *Trifolium*, and number of flower heads for *Fagopyrum*), number of stems/tillers of the barley (determines ear density), and at the end of the experiment the barley dry biomass (dried at 40°C for 3 days) and seed yield was collected. For the insect community, we identified all aphids to species and counted abundance for each plant. Natural enemies observed on the different plants were identified at least to family but to genus where possible. Ladybirds were split into ladybird larvae and ladybird adults (but eventually combined for analyses). Parasitoid wasps were identified through the mummies that are formed when the wasp develops within the aphid host; the aphid mummy color indicates the genus of parasitoid wasp. A set of aphids and parasitoids were collected to confirm identification (either by morphology or molecular methods using universal COI primers following Gossner et al. (2016)).

### 2.5 Data analysis

To compare the 2017 (16 observation days) and 2018 (8 observation days) data sets for aphid numbers, we used peak aphid population size (i.e., the maximum number of aphids counted during one observation day). Aphid peak population is an informative variable since timing of arrival and growth rates can differ among species, due to preceding weather and the ability of natural enemies to control outbreaks (among other factors). Here, it allowed us to compare the data across the years, species, and treatments. We also analyzed the data across the season using repeated measures, but it did not provide further information than considering cumulative factors and thus is not presented.

All data were analyzed in R 3.6.3 (R Core Team, 2020) in R Studio 1.2.5033 (RStudio Team, 2020). We used generalized linear models with quasi‐Poisson error on count data (aphid number, natural enemy abundance, tiller number, flower number) and linear models with normal error distributions for continuous data (natural enemy diversity, barley biomass, barley relative growth rate, seed mass) to measure the effects of year, barley cultivar, and flower treatment (flower richness as well as all treatments that include flower identity). All models contained the experimental blocking factors indicating the replicate (Block) and distance from the meadow border (row). Further, covariates were used where necessary, predominantly barley biomass to account for plant size, and total number of unwinged aphids to account for aphid resource.

We also used Structural Equation Modelling (SEM) in the R package “piecewise” (Lefcheck, 2016), building two models for each year using linear mixed effect models with block and row as random effects. The first model aimed to give a general overview of the effects of the individual flowers on hosting nonbarley aphids, recruiting parasitoids and predators, and the effect on the maximum population of the main aphid species. The second model tests the effect of each aphid species across each flower on establishing the different natural enemy populations with resulting effect on the natural enemy community of barley and aphid maximum population sizes. Parasitoid wasps were separated by species, but generalist predators were grouped together due to low abundance of individual species. Model fit was evaluated using Fisher's C statistic with each presented model reproducing the data well (*p* > .05).

## 3 RESULTS

### 3.1 Aphid and natural enemy species

In 2017, we counted 1694 winged aphids and 25,057 unwinged aphids over 16 days of data collection (twice per week). Almost half of the unwinged aphids counted (12,307) were observed on the barley plants, including 7673 *Sipha elegans*, 3079 *Diuraphis noxia*, 1506 *Rhopalosiphum padi* individuals for which we focus on for the main analyses; additionally, 49 *Sitobion avenae* were observed. The remaining aphids were observed on the flower plants including 7267 *Aphis fabae* (on *Fagopyrum*, *Trifolium*, and *Vicia*), 1842 *Acyrthosiphon pisum* (on *Trifolium* and *Vicia*), 2086 *Macrosiphum euphorbiae (on Fagopyrum)*, and 1555 *Megoura viciae* (on *Vicia*).

In 2018, over 8 days of data collection (once per week), we counted 3755 winged aphids and 20,259 unwinged aphids. Of these, 4369 unwinged aphids were counted on the barley plants including 548 *S*. *elegans*, 1159 *D*. *noxia*, and 951 *R*. *padi* individuals for which we can compare across years. We also counted 1274 *Sitobion avenae* and 437 *Metopolophium dirhodum* aphids on barley that we refer to in certain analyses but cannot compare back to 2017 due to low numbers observed. The remaining aphids were on the flower plants, including 15,292 *Aphis fabae* (on *Fagopyrum*, *Trifolium*, and *Vicia*), 322 *Acyrthosiphon pisum* (on *Trifolium*), 181 *Macrosiphum euphorbiae (on Fagopyrum)*, and 95 *Megoura viciae* (on *Vicia)*.

The natural predator community across both years (all species were observed directly consuming aphids on the plants) included 66 ladybird larvae and adults (Coleoptera: Coccinellidae), 51 lacewing larvae (*Chrysoperla carnea* Neuroptera: Chrysopidae), 23 syrphid larvae (Diptera: Syrphidae), and 24 soldier beetles (*Rhagonycha* sp. Coleoptera: Cantharidae). We also observed parasitoid mummies (hardened shell of a parasitized aphid) from three groups of primary endoparasitoids (349 *Praon* sp. 148 *Aphidius* sp. (only in 2018) and 42 *Aphelinus* sp.), hyperparasitism was not investigated. Molecular barcoding of a subset of parasitized aphids collected in 2018 from barley plants (as part of a related project) identified the dominant parasitoid species as *Praon volucre* and *Aphidius rhopalosiphi*.

**FIGURE 1:**
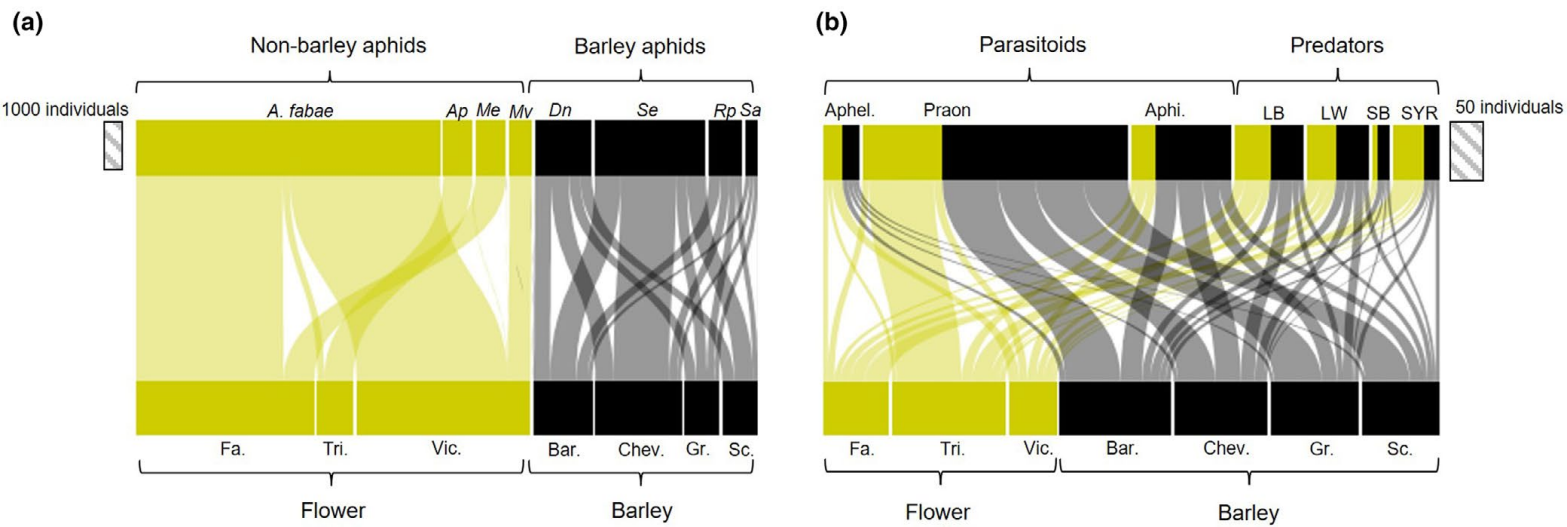
Quantitative food webs for (a) plants to aphids and (b) plants to natural enemies. Flower plants are shown in yellow with their contribution to overall aphid and natural enemy abundances. Flower: Fa. *Fagopyrum esculentum*, Tri. *Trifolium pratense*, Vic. *Vicia faba*, Barley varieties: Bar. Barke, Chev. Chevallier, Gr. Grace, Sc. Scarlett. Aphids: A. fabae *Aphis fabae*, Ap *Acyrthosiphon pisum*, Me *Macrosiphum euphorbiae*, Mv *Megoura viciae*, Dn *Diuraphis noxia*, Se *Sipha elegans*, Rp *Rhopalosiphum padi*, Sa *Sitobion avenae*. Parasitoids: Aphel. *Aphelinus* sp., *Praon* sp, Aphi. *Aphidius* sp. Predators: LB ladybirds (Coccinellidae), LW lacewing larvae (Chrysopidae), SB soldier beetle (Cantharidae), SYR syrphid/hoverfly larvae (Syrphidae). Data show 2017 and 2018 data combined

**FIGURE 2:**
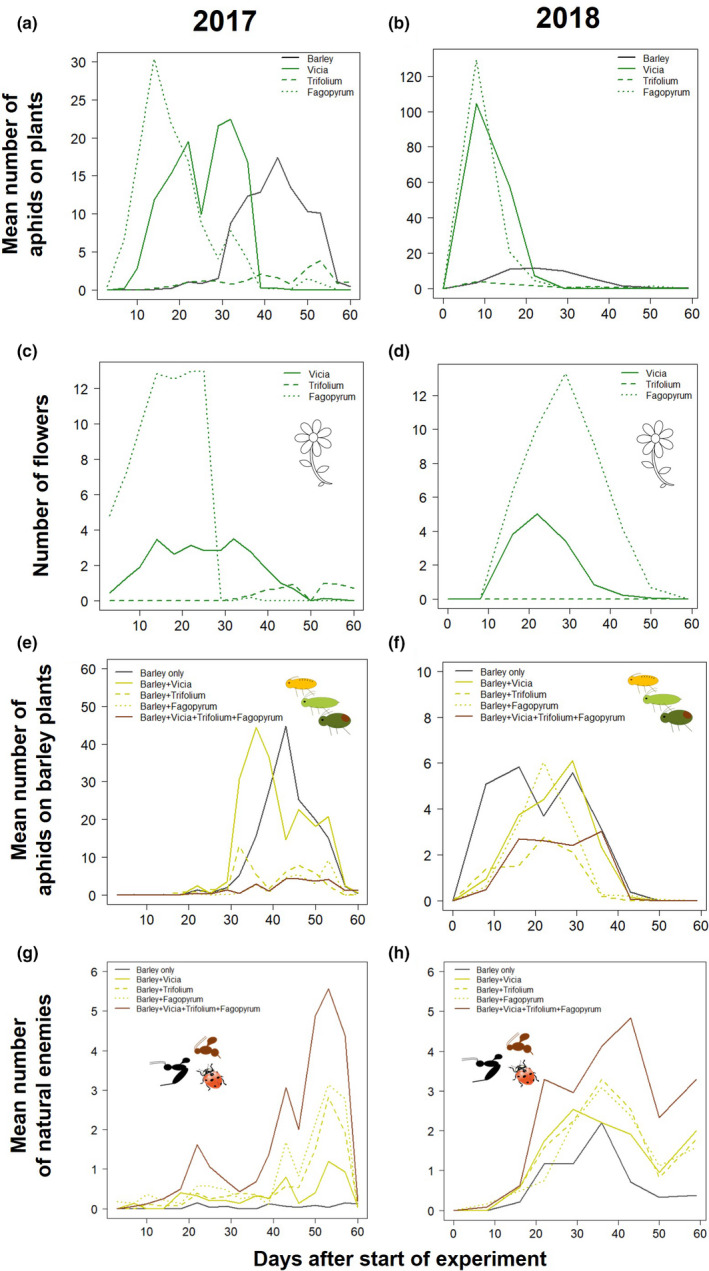
Colonization of plants by aphids, natural enemies, and the flower resource available over the experimental periods in 2017 and 2018. Aphid colonization of all plants (a, b), with the number of flowers in each treatment (c, d), the number of aphids on barley (e, f) and abundance of natural enemies (g, h) across flower treatments

**FIGURE 3:**
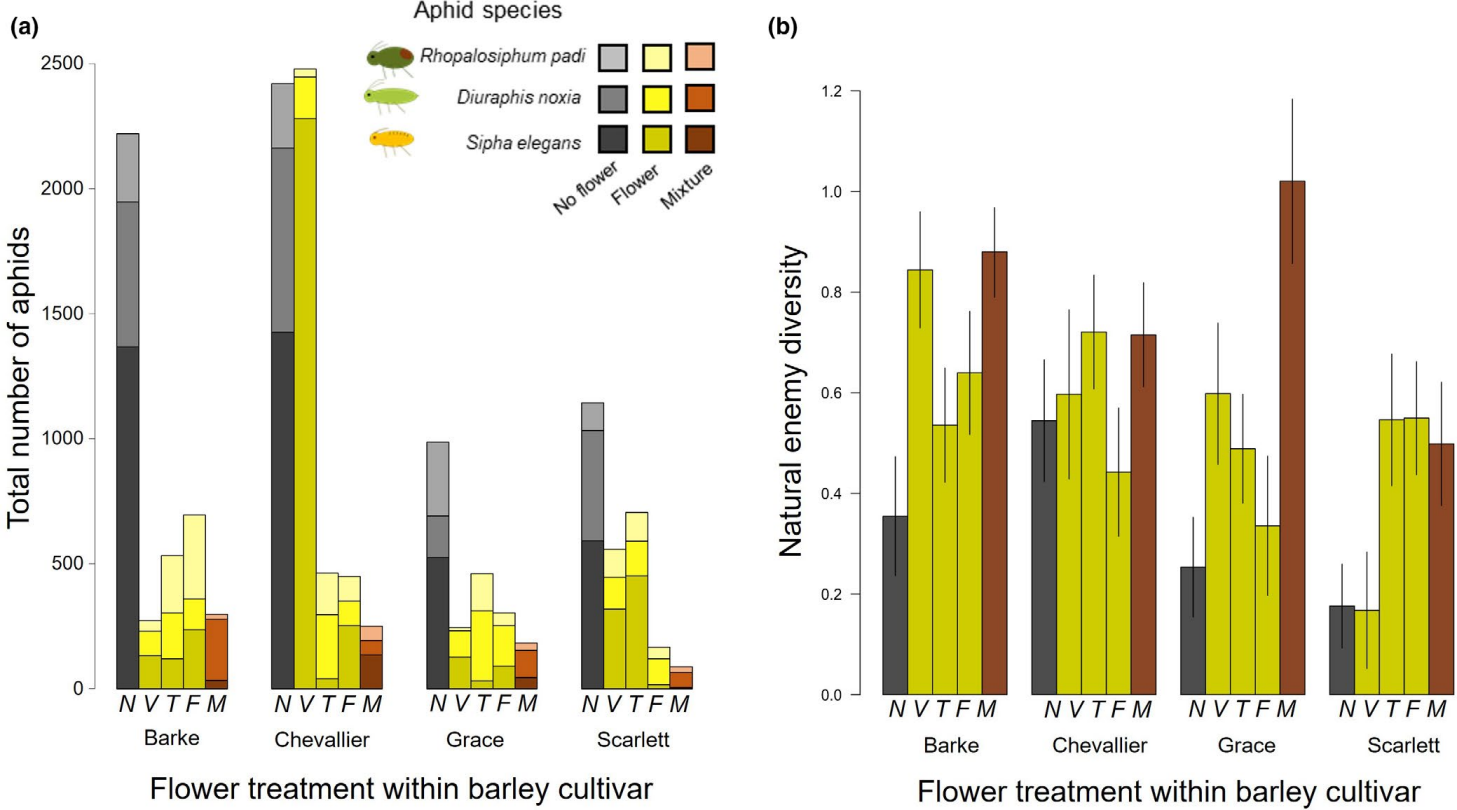
Aphid abundance and natural enemy diversity. (a) Total number of aphids, stacked by aphid species and (b) natural enemy diversity across the four barley cultivars and five flower treatments (N: No flower, V: *Vicia faba*, T: *Trifoilum pratense*, F: *Fagopyrum esculentum*, M: Mixture of the three flowers). Error bars show ± 1*SE*

There was no overlap between aphid species on the flower plants and those of the barley (Figure 1a), while natural enemies were shared across all flower species and barley varieties (Figure 1b). Aphids first colonized *Fagopyrum* and *Vicia* plants, before colonizing *Trifolium* and barley plants (Figure 2a,b). *Fagopyrum* provided the highest number of flower heads but they peaked early and then died off as the plants set seed, while *Vicia* produced fewer flowers for a longer period of time and *Trifolium* flowered later (with few flowers produced in 2018) (Figure 2c,d). The aphids on barley arrived earlier and persisted longer in 2018 than in 2017, but peak population sizes were higher in 2017 (Figure 2e,f); this is likely due to a 2‐week delay in the plants going outside in 2018 (due to stormy weather) and subsequently two high rainfall events (days 14 and 30) in 2018 that restricted aphid growth rates and reduced peak population sizes. Aphid maximum population size varied across barley cultivars for two species (*S*. *elegans F*_3,239_ = 2.61, *p* = .052; *D*. *noxia F*_3,239_ = 0.81, *p* = .488; *R*. *padi F*_3,239_ = 2.85, *p* = .039); in general, aphids were more abundant on Barke and Chevallier barley cultivars than Scarlett and Grace, particularly when no flower was present (Figure 3a).

### 3.2 Effects of plant species richness and plant identity on aphids and natural enemies

Peak population sizes of the three main barley‐aphid species were reduced on the barley plants when flowering plants were present, and the effect was strongest when all flowers were present (Flower richness: *S*. *elegans F*_2,239_ = 15.86, *p* < .001; *D*. *noxia F*_2,239_ = 10.14, *p* < .001; *R*. *padi F*_2,239_ = 8.11, *p* < .001). Flowering plant species identity had strong effects on the reduction of aphid numbers, but this effect depended on the barley aphid species (Table 1; Figure 3a), with limited interaction effects across the different barley cultivars (Table 1; Figure 3a). In particular, we found that fewer *S*. *elegans* aphids were observed on barley plants next to *Fagopyrum* or *Trifolium* plants, while those next to *Vicia* hosted similar numbers to control barley plants next to no flower (Figure 3a). In general, we observed more *D*. *noxia* aphids on barley next to no flowers (Table 1; Figure 3a). However, in 2017, there was a slightly stronger impact of the flowers on *D*. *noxia* aphids than in 2018 (Table 1), with fewest aphids on barley next to *Fagopyrum* in 2017, and next to *Trifolium* in 2018. Lastly, *R*. *padi* aphids on barley were reduced next to *Vicia* in both years (Figure 3a), but the impact of the other flowers was opposite across the two years and further dependent on the barley cultivar (Year × barley × flower; Table 1). The main factors driving this interaction included a lower number of aphids on barley with no flower in 2018 for all cultivars except Grace, higher number of aphids in 2018 on Scarlett barley next to *Vicia*, and higher numbers of aphids in 2018 on Barke barley next to *Fagopyrum*.

**TABLE 1:** Summary of the effect of flower treatment across barley cultivars and experimental years on aphid, natural enemies, and plants

	Year		Barley cultivar		Flower treatment^a^		Year × Barley		Year × Flower		Barley × Flower		Year × Barley × Flower	
Aphids														
*Sipha elegans*	0.56	0.455	2.61	0.052	**10.76**	**<0.001**	–	–	–	–	–	–	–	–
*Diuraphis noxia*	1.33	0.250	0.83	0.477	**5.78**	**<0.001**	–	–	2.31	0.059	–	–	–	–
*Rhopalosiphum padi*	3.41	0.066	**2.85**	**0.039**	**4.88**	**<0.001**	2.29	0.080	1.71	0.149	**1.59**	**0.010**	**2.20**	**0.013**
Natural enemies														
Diversity	**7.45**	**0.007**	2.25	0.084	**7.04**	**<0.001**	–	–	–	–	**1.80**	**0.050**	–	–
Abundance	**43.12**	**<0.001**	0.87	0.458	**12.40**	**<0.001**	–	–	**3.73**	**0.006**	**2.10**	**0.018**	–	–
Parasitoid abundance	**43.79**	**<0.001**	0.96	0.410	**2.64**	**0.035**	–	–	–	–	**2.84**	**0.001**	–	–
Predator abundance	**10.68**	**0.001**	0.27	0.846	**15.77**	**<0.001**	–	–	**7.14**	**<0.001**	–	–	–	–
Plant														
Plant RGR (height)	**335.23**	**<0.001**	**14.15**	**<0.001**	**4.27**	**0.040**	**2.89**	**0.037**	0.01	0.951	0.28	0.839	**3.58**	**0.015**
Plant biomass	**6272.30**	**<0.001**	**64.82**	**<0.001**	**6.56**	**0.011**	**33.93**	**<0.001**	–	–	–	–	–	–
Tiller number	**1149.50**	**<0.001**	**38.55**	**<0.001**	2.44	0.12	**9.20**	**<0.001**	–	–	–	–	–	–
Yield (seed mass)	**17.75**	**<0.001**	**32.00**	**<0.001**	**6.48**	**0.012**	**11.76**	**<0.001**	–	–	–	–	–	–

Note

Values in bold are significant at *p* < .05.

^a^ Flower treatment includes plant identity for aphids and natural enemies but only presence/absence of a flower for plant traits. GLMs with quasi‐Poisson errors used for count data (aphid models, natural enemy abundances, tiller number) and LMs for continuous data (natural enemy diversity, plant RGR, biomass, and yield).

The diversity and abundance of natural enemies was increased in treatments containing flowers, with the mixed flower combination further increasing abundance and diversity (Table 1; Figure 3b). Notably, in 2018, we observed no *Aphelinus* sp. parasitoid mummies on barley that was alone yet they were abundant on barley next to flowers. There was also some variation in the response to the flower treatment across barley cultivars (Figure 3b; Table 1); for example, while the flower mixture recruited high natural enemy diversity for all barley varieties, *Vicia* increased natural enemy diversity for cv. Barke plants to levels similar to the flower mixture but decreased diversity for cv. Scarlett plants. Aphid peak population sizes were differentially influenced by the overall natural enemy diversity and abundance of predators or parasitoids. A reduction in peak population size of *S*. *elegans* aphids was associated with increased parasitoid abundance (*F*_1,239_ = 6.28, *p* = .013), while *R*. *padi* aphids were reduced by increased predator abundance (*F*_1,239_ = 13.93, *p* < .001). Both *D*. *noxia* and *R*. *padi* aphids were reduced by overall natural enemy diversity (DN: *F*_1,239_ = 2.99, *p* = .085; RP: *F*_1,239_ = 7.34, *p* = .007) (explored further in SEM community analysis section).

### 3.3 Plant growth and seed mass

**FIGURE 4:**
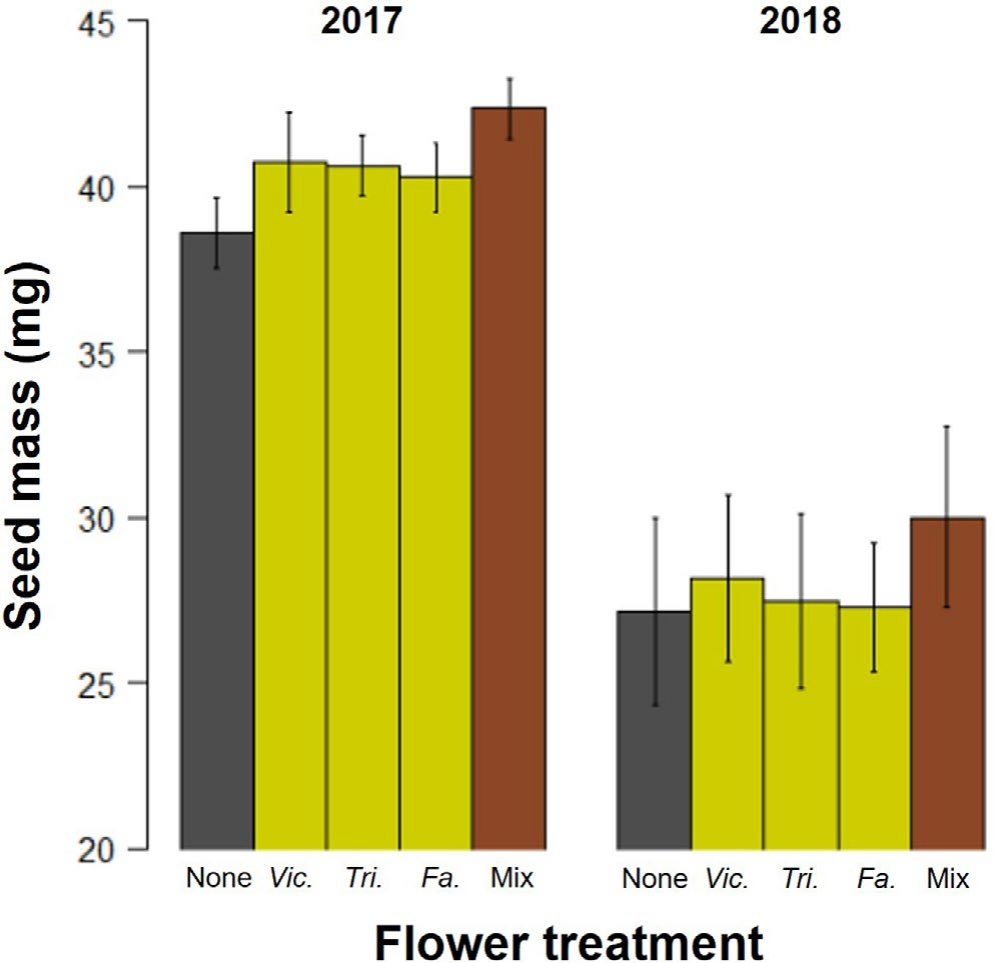
Barley seed mass (mg) across the two experimental years and flower treatments. Results averaged across barley cultivar. Error bars show ±1*SE*

Seed mass was increased for barley plants next to any flower compared to no flower (Table 1; Figure 4); however, flower identity did not alter seed mass (*F*_2,149_ = 0.61, *p* = .922; Figure 4) or other traits. Similarly, barley plants next to flowers (presence/absence) experienced a higher relative growth rate, but a decrease in final biomass, and no change in the number of tillers (stems) (Table 1). All plant traits varied across the years also dependent on barley cultivar (two‐way interactions; Table 1), while for plant relative growth rate (height), this interaction also included flower treatment (Table 1). These effects were not driven by the increased natural enemy diversity on mixtures, abundance of aphids, or natural enemy groups (all *p* > .05).

### 3.4 Community assembly and aphid suppression

**FIGURE 5:**
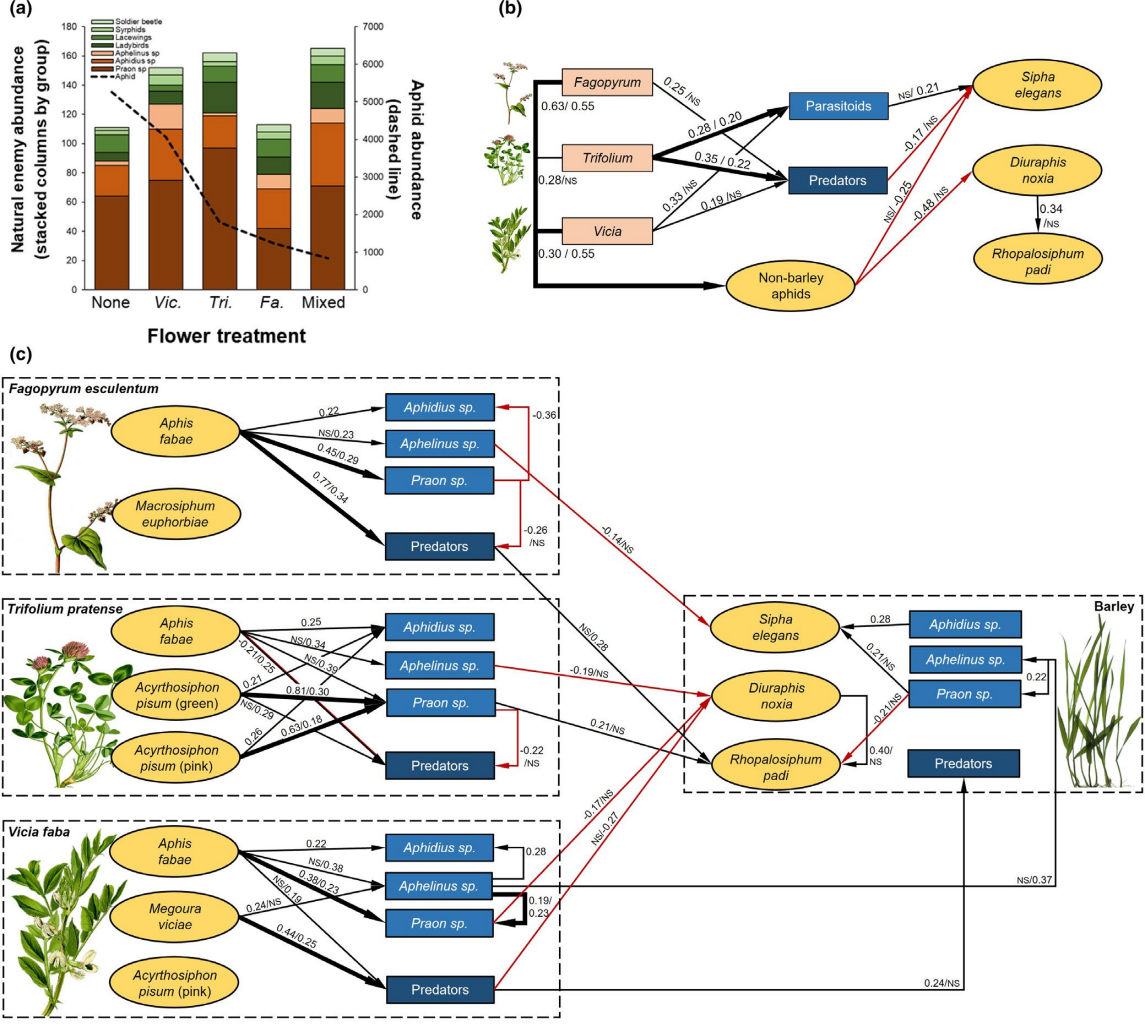
Outcome of interactions on insect abundance and the underlying networks. (a) Natural enemy abundance (brown color for specialist parasitoid wasps, and green for generalist predator species) across flower treatment with the dotted line showing mean barley aphid abundances, (b) the simple network of flower treatment effect on nonbarley aphids, natural enemies, and barley aphid populations, and (c) the complex network of natural enemies associated with different nonbarley species on the flowers. In the network figures (from SEM analyses), the arrows show the direction of effect and two numbers show the strength of effect across the two years (2017/2018), only significant links are shown. Red arrows indicate a negative association and black a positive association

There was a time‐lag from initial aphid colonization until the natural enemy community was established (~10 days) and natural enemy numbers remained highest on the mixed flower treatments for the duration of the experiment in both years (Figure 2g,h). The abundance of parasitoids and predators varied across the flower treatments with more parasitoids on *Vicia* and *Trifolium*, while predators were most abundant on *Trifolium* and *Fagopyrum* (Table 1; Figure 5a). We used structural equation modeling to further understand the impact of direct and indirect interactions among the flower treatments, nonbarley aphids, and natural enemy species on aphid population sizes (Figure 5b,c). Barley cultivar had no significant effects (*p* > .05) and thus was removed from the models. Overall, the presence of *Trifolium* and *Vicia* had the strongest effect on increasing the total number of parasitoids and predators, while *Fagopyrum* plants recruited more predators (Figure 5b). However, the total number of parasitoids and predators at the level of the treatment (i.e., barley plant plus the flowers) had minimal effect on the aphid population sizes, whereas the number of nonbarley aphids had stronger negative effects across the years. Thus, it is not simply the total number of natural enemies that reduces aphid populations, but via indirect effects via the nonbarley aphids established on the noncrop plants.

We explored this further to understand the importance of specific interactions with the nonbarley aphids at the level of flower identity (Figure 5c). Here, we found that the identity of the aphid species on each flower species was important. *Aphis fabae* aphids are highly polyphagous and were abundant on all three flower species across both years (*Fagopyrum n* = 10,884, *Trifolium n* = 528, *Vicia* n = 11,147). On all three flower species, these aphids benefitted the establishment of parasitoid communities (particularly *Praon* sp. on *Fagopyrum* and *Vicia*) and recruitment of predator communities (particularly on *Fagopyrum*) (Figure 5c). The other aphid species on *Fagopyrum* did not contribute to natural enemy community establishment (*M*. *euphorbiae* aphids, *n* = 2267). On *Trifolium*, both the green and pink color morphs of pea aphids (*A*. *pisum*, *n* = 1292 green and *n* = 841 pink) helped to establish parasitoid communities (particularly *Praon* sp.), while only the green morph increased predator abundance (Figure 5c). Only pink pea aphids (no green ones) were observed on *Vicia* in very low numbers (*n* = 31) indicating a high host preference for *Trifolium* in this experiment; these aphids did not promote the natural enemy community on *Vicia*. The large *Megoura viciae* aphid with distinct black legs (*n* = 1650) was associated with higher number of predators on *Vicia* plants in both years and helped to establish *Aphelinus* sp. parasitoids in 2017 (Figure 5c).

Parasitoids on all three flower species and predators on *Vicia* directly reduced the number of aphids on the barley plants, while only the population of *Praon* sp. parasitoids on barley plants themselves contributed to aphid control (Figure 5c). *Aphelinus* sp. parasitoids and predators on *Vicia* directly contributed to establishing the natural enemy community on barley plants, but this did not translate into a reduction of aphid population sizes. Some negative associations among the natural enemy groups indicated competition, for example, the abundance of *Praon* sp. parasitoids reduced the abundance of general predators on *Fagopyum* and on *Trifolium*. In contrast, there was some potential for facilitation observed between *Praon* sp. and *Aphelinus* sp. parasitoids on barley and *Vicia* plants (Figure 5c).

## 4 DISCUSSION

Our results clearly show that providing natural enemies with a selection of flowering plants can reduce aphid population sizes on potted barley plants through an increase in natural enemy diversity. We further show that the benefit of multiple flowers for aphid suppression is a result of numerous weaker interactions between different flower, aphid, and natural enemy species, rather than a few dominant interactions. Each flower species recruited a different community of nonbarley aphids that, in turn, varied in their ability to establish the natural enemy populations and subsequently the ability to reduce barley aphid populations. This resulted in the mixture of the three flowering plants recruiting the highest diversity and abundance of natural enemies, with subsequent greatest reduction in aphid populations in both experimental years. The contribution of each individual flower species to the overall community also shows that this is not just a result of increased flower density, but rather of flower identity, even if the aphid suppression outcome is similar. The general patterns across the two years were also similar, while the strength or significance of some interactions varied. Since the system relies on few dominant direct interactions but many weaker indirect interactions, this allows for an overall greater potential for aphid suppression across different years and potentially variable environments (van Veen et al., 2006).

That diversity begets diversity is not a new finding (Palmer & Maurer, 1997; Scherber et al., 2010; Snyder & Tylianakis, 2012), but our work shows that plant identity is likely an important factor for effective biocontrol in crop systems. While we hypothesized that the flowers themselves would be important for general natural enemy recruitment, as they offer a nectar resource for many of the adult parasitoid/predators, it was the variety of nonbarley aphids on these plants that was key for aphid suppression. Our modeling approach showed that the different plants were establishing different natural enemy communities, and consequently, the mixed flower treatment experienced stronger aphid suppression due to using an additive experimental design. In particular, *Aphis fabae* aphids on *Fagopyrum* recruited the most generalist predators, while *Acyrthosiphon pisum* aphids on *Trifolium* had the strongest effect in recruiting *Praon* sp. parasitoids. Almost every aphid species on each plant played a role in natural enemy recruitment. Complementarity in the system allows each species to perform its own function by establishing a different community through occupying different feeding, spatial or temporal niches (Snyder, 2019; Snyder & Tylianakis, 2012). However, simply increasing plant species or functional diversity may not result in the promotion of these beneficial interactions (Hatt et al., 2017).

The aim of floral plantings is to establish natural enemy populations that then spill over to the crop plants (Blitzer et al., 2012; Morandin & Kremen, 2013). This must occur for the duration of the cropping season otherwise natural enemies may experience competition for prey resources that leads them to move away from the area (Snyder & Tylianakis, 2012). Alternatively, if the floral planting hosts too much prey resource, the natural enemies will never spill over into the crop (Kremen et al., 2019). In some circumstances, increasing the prey resource not only stops the natural enemy spill over but can also build trophic complexity in the system. This can result in reduced pest suppression due to “ecological redundancy” among different species, reducing the effect of increased biodiversity on pest control (Snyder, 2019). It is clear there is a trade‐off between recruiting sufficient noncrop prey populations to establish a diverse natural enemy community, and the noncrop prey persisting at high abundances for the whole season trapping natural enemies within the noncrop plants. In our experiment, the *Fagopyrum* and *Vicia* flower resource and alternative prey aphids decreased just before the peak of barley aphids, providing an ideal situation for movement of natural enemies to the barley. The addition of *Trifolium* plants benefitted the system toward the middle and end of the experiment, with strong effects on natural enemies despite low nectar resource. We observed stronger aphid suppression in the mixed flower treatment due to the variation in flowering time, establishment of nonbarley aphids and recruitment of natural enemies across the different plants. Similar results have been found in field studies on tailored flower strips to control potato aphids (Tschumi et al., 2016) and leaf beetles (Tschumi et al., 2015). However, floral planting studies often focus on how flowering plants can attract natural enemies (Hatt et al., 2019) rather than the whole ecological system. We suggest that a focus on the flowers as a supporting system for the beneficial pest–natural enemy interactions is key.

We did not detect strong links between the natural enemies on the flower plants with natural enemy populations on the barley plants themselves, that is, establishment of natural enemy populations on the crop itself. Yet negative interactions linking the natural enemies on the flowering plants and the aphid population sizes on barley suggest the natural enemies moved back and forth between then plants. The high mobility of the natural enemies and close proximity of the barley and flower plants due to the small‐scale setup of our pot experiment benefitted this. Floral plantings can act as shelters for the predators and parasitoids during periods of rest by improving the microclimate, protection from intraguild predation, and the previously discussed provision of alternative prey (reviewed by Gontijo, 2019). Distance to flower strip is a commonly significant variable for pest control effectiveness (Albrecht et al., 2020) and habitat preferences may explain why natural enemies stay closer to the edges rather than move further into the field. The distances used in the current experiment are obviously much shorter than those in the field and therefore further work is needed to optimize natural enemy movement into the crop. This also shows the potential for using these plants as an intercrop rather than as flower strips on the border of fields, allowing for more in‐field benefits. Identifying what is needed by the various natural enemies in an agroecosystem can help to identify those flower species that can provide these requirements, for example, by using simulations based on empirical data (Bianchi & Wäckers, 2008).

Under integrated pest management schemes, the ecology of the system drives the decision making for pest control strategies. By knowing the common pest insect, noncrop (resource) insects, and natural enemies in a given area, we can begin to design effective flower mixtures to enhance natural pest control. For example, Gontijo et al. (2018) found that nocturnal biocontrol of aphids by predators was hampered by intercropping with a plant that benefitted overall abundance of natural enemies. Thus, flowers must provide the resource when needed but not hinder pest control efforts by other species in the system. Choosing plants with variable growth rates, flowering times, and growth habits can promote the establishment of a diverse natural enemy community.

In conclusion, we identified many weak interactions that together led to stronger suppression of aphids on potted barley plants that were grown next to a flower with even stronger aphid suppression when all flowers were present. The flowers were chosen for their previous use in flower strips, as banker plants and potential for intercropping. In this system, *Fagopyrum* grew fast, flowered before the others and established an early nonbarley aphid population that recruited initial populations of natural enemies, followed by *Vicia*, which flowered soon after. Lastly, *Trifolium* plants flowered late but rather than flower resource, they provided additional alternative prey resources and shelter for the natural enemies in the latter part of the experiment. By understanding the colonization of various crop plant and flower plants by aphid species and the shared natural enemies, we can begin to tailor floral plantings to enhance biological control effectiveness in field systems.

## AUTHOR CONTRIBUTION

**Sharon E. Zytynska:** Conceptualization (lead); Formal analysis (lead); Funding acquisition (lead); Investigation (equal); Methodology (equal); Visualization (lead); Writing – original draft (lead); Writing – review & editing (equal). **Moritz Eicher:** Investigation (equal); Methodology (equal); Writing – review & editing (supporting). **Robin Fahle:** Investigation (equal); Methodology (equal); Writing – review & editing (supporting). **Wolfgang W. Weisser:** Conceptualization (supporting); Writing – review & editing (equal).

## Data Availability

Data are available at https://doi.org/10.5061/dryad.pk0p2ngps (Zytynska et al., 2021).
